# Refining Viral Production Estimation

**DOI:** 10.1111/1758-2229.70258

**Published:** 2025-12-14

**Authors:** Hisham M. Shaikh, Jonas Van den Bremt, Lisa Schellenberg, Salvador J. Fernández Bejarano, Maarten De Rijcke, Corina P. D. Brussaard

**Affiliations:** ^1^ Research Department Flanders Marine Institute (VLIZ) Ostend Belgium; ^2^ Department of Biochemistry and Microbiology, Faculty of Sciences Ghent University (UGent) Ghent Belgium; ^3^ Department of Marine Microbiology and Biogeochemistry Royal Netherlands Institute for Sea Research (NIOZ) Den Burg the Netherlands; ^4^ Department of Freshwater and Marine Ecology, Institute for Biodiversity and Ecosystem Dynamics (IBED) University of Amsterdam Amsterdam the Netherlands; ^5^ Data Centre Division Flanders Marine Institute (VLIZ) Ostend Belgium

**Keywords:** bacterial growth, lysogeny, lytic, marine viruses, mitomycin C, mortality, VIPCAL, viral production assay, viralprod

## Abstract

Viral‐mediated bacterial mortality and the prevalence of lysogeny are two key parameters for understanding the role of viral activity in aquatic ecosystems. The viral production assay is most commonly used to assess these parameters, with lytic and mitomycin C‐induced viral production rates prevalently extracted using the linear regression or increment‐based (VIPCAL) approach. A literature survey shows that 64% of the 89 viral production studies used the linear regression approach for lytic and 48% employed VIPCAL for lysogenic viral production rates. Our comparative evaluation highlights significant differences between these two approaches of estimating viral production rates. To refine estimations, we enhanced VIPCAL to VIPCAL‐SE by incorporating standard error of the means to rigorously identify maxima–minima pairs, accounting for biological and ecological variabilities between replicates. We also included a bacterial net generation time endpoint to reduce estimation bias due to potential secondary infections, particularly relevant in more productive ecosystems. VIPCAL‐SE is now available as a part of the *viralprod* R package and provides an opportunity for further standardisation in the field of aquatic viral ecology.

## Graphical abstract

Viral production assays are generally employed to decipher viral‐mediated bacterial mortality rates and prevalence of lysogeny in aquatic ecosystems. Commonly used analytical approaches—linear regression and VIPCAL—often provide significantly different estimates. This study refines viral production estimations by incorporating standard errors and bacterial net generation time to constrain the incubation period.

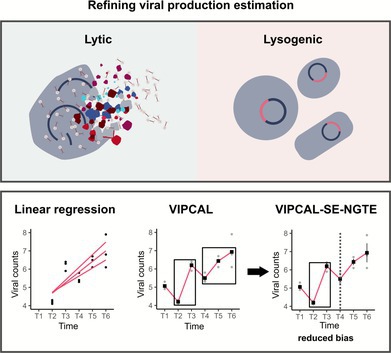

## 1 Introduction

Constituting the largest share of the living biomass in the world's oceans, marine microorganisms drive critical biogeochemical cycles through diverse and interdependent metabolic roles (Suttle 2007; Falkowski et al. 2008; Bar‐On et al. 2018). They face constant selective pressure from environmental factors and interspecific interactions like allelopathy, symbiosis or mortality (Strom 2008). Successful interactions with lytic viruses result in the production of viral progeny and subsequently the death of the unicellular host cell. With an estimated 10^28^ lytic viral infections occurring in the world's ocean every day, the 10^9^ tons of organic carbon released is readily degraded by heterotrophic bacteria (Suttle 2007; Middelboe et al. 1996; Brussaard et al. 2005; Lønborg et al. 2013). The lysed organic matter fuels the microbial loop, thereby diverting energy and matter away from higher trophic levels (Wilhelm and Suttle 1999; Lønborg et al. 2009; Evans et al. 2021). Furthermore, through host selectivity and the consequent host–virus arms race, viruses shape the structure and functions of bacterial communities.

Temperate viruses can enter a lysogenic cycle, during which they integrate into the host genome as ‘prophages’ and replicate passively along with the host cell without causing immediate cell death (Payet and Suttle 2013; Howard‐Varona et al. 2017; Tuttle and Buchan 2020). Prophages contribute to genetic evolution and may provide competitive advantages to marine microbial lysogens (bacteria containing a prophage) by carrying auxiliary metabolic genes involved in processes such as photosynthesis, carbon metabolism and virulence factors like endotoxin/exotoxin genes (Howard‐Varona et al. 2017; Schwalbach et al. 2004; Fortier and Sekulovic 2013; Yi et al. 2023). Exposure to environmental stressors (UV radiation, antibiotics, nutrient shifts, quorum sensing molecule concentrations, temperature changes, pH fluctuations, among others) can trigger prophage induction, where a prophage excises itself out of the bacterial chromosome and enters into the lytic cycle (Cochran et al. 1998; Weinbauer and Suttle 1999; Chu et al. 2011; Silpe and Bassler 2019; Henrot and Petit 2022). Human activities—such as pollution, climate change and nutrient enrichment—can exacerbate these stressors, influencing viral–host interactions and the stability of microbial communities and their functioning (Danovaro et al. 2011).

Viral‐mediated bacterial mortality and the prevalence of lysogeny are crucial ecological parameters for understanding viral ecology in aquatic systems (Jiang and Paul 1998; Mojica and Brussaard 2020). According to the global Virus Oceanography Database (gVOD), the viral production assay (Weinbauer and Suttle 1999, 1996) is the most popular method to estimate viral‐mediated bacterial mortality in diverse aquatic bacterial communities (Xie et al. 2021). The viral production assay is based on the release of viruses produced from infections that occurred prior to the start of the assay. To determine the virus production rate, viruses in the original seawater are washed out using a 0.2 μm pore‐size filter, typically by tangential flow filtration (Winget et al. 2005). The resulting virus‐reduced seawater sample is then incubated in replicates, kept in the dark at in situ temperature, and subsampled over time to enumerate viruses released from cell lysis due to earlier infections (Winget et al. 2005; Wilhelm et al. 2002; Weinbauer et al. 2010). The reduction of viruses from the original seawater sample not only reduces the contact between viruses and their bacterial hosts, preventing secondary infections, but is also necessary for quantifying significant increases in virus production.

**FIGURE 1:**
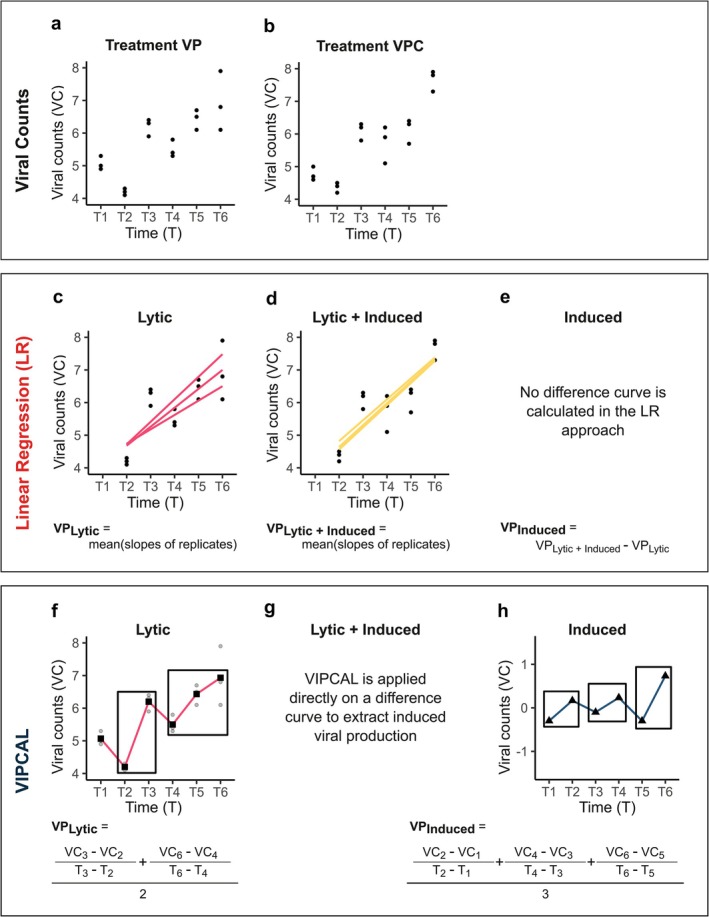
Schematic representation of the two major prevalent data analysis approaches of viral production assay: The linear regression and VIPCAL. Viral counts (VC; in viruses mL^−1^) are plotted over time (*T*; in h) for unamended incubation, VP (a) and mitomycin C‐treated incubation, VPC (b) treatments. The linear regression approach selects a period of productivity (here, *T*_2_–*T*_6_) and calculates lytic (c) and combined lytic and induced viral production rates (d) by averaging the mean of the slopes of viral counts (VC) over time. In the linear regression approach, induced viral production rate is calculated by subtracting lytic from the combined production rate (e). VIPCAL computes the lytic viral production rate (f) by analysing changes in viral counts over defined time intervals. However, it is not directly applied to the induced + lytic treatment (VPC) to estimate induced viral production (g). Instead, induced production is inferred by applying the method to a difference curve (h) that is calculated by subtracting averaged VP viral counts from VPC per time point, capturing fluctuations indicative of induced viral production.

The data analysis of the viral production assay is thus far performed using either the linear regression (Winget et al. 2005) or the increment‐based approach (Weinbauer et al. 2002) (Figure 1). The linear regression approach calculates the net viral production rate by averaging the slopes of first‐order linear regressions derived from viral counts over time for each replicate (Figure 1c,d) (Noble and Fuhrman 2000). This method relies on a single continuous range, fitting fluctuating viral counts over time. Often, a specific period of productivity is selected, based on the potentially delayed release of viruses and/or virus production leveling off towards the end of the incubation (Figure 1c,d). While the linear regression approach smooths out fluctuations in viral counts over time by calculating a slope, the increment‐based approach does not take periods of decline in viral counts into account when estimating viral production rates (Figure 1f,h). Fluctuations in viral abundances may result from changes in latent periods, adsorption rates or new infections. The increment‐based analysis was developed based on studies by Weinbauer et al. (2002) who proposed an alternative analysis to linear regression where the net viral production rate is measured as the difference between averaged maximum and minimum viral counts over time. Accounting for fluctuations in viral counts, Winter et al. (2004) updated the approach to allow multiple maximum–minimum pairs, and Luef et al. (2009) standardised the approach into the online tool VIPCAL (Viral Production Calculator; https://nuhagphp.univie.ac.at/vipcal/). The increment‐based VIPCAL approach provides less conservative estimates of viral production compared to linear regression but may be more susceptible to overestimations due to variance among replicates and time points, as it averages viral counts across replicates for each time point, potentially amplifying the effect of outliers.

In addition to determining lytic viral production by aquatic prokaryotes, the assay can be used to estimate the prevalence of lysogeny by adding the antibiotic mitomycin C as the inducing agent to trigger prophages into entering the lytic cycle (Weinbauer and Suttle 1999; Sekiguchi and Takagi 1960; Evans et al. 2009; Evans and Brussaard 2012). For the linear regression approach, the difference in viral production rates between mitomycin C‐treated (VPC) and untreated (VP) series provides the mitomycin C‐induced viral production rate used to estimate the prevalence of lysogeny (Figure 1e). Alternatively, VIPCAL estimates the occurrence of lysogeny from a difference curve that is calculated by subtracting averaged viral counts per time point of VP from VPC treatments per time point (Figure 1h). While VIPCAL offers generally higher reproducibility as an online tool, the lack of inspection and risk of overestimating production rates influence the confidence in viral production rate estimations. Both approaches have strengths and limitations, yet it is unclear how these approaches compare in estimating viral production rates.

The duration of the assay and subsampling frequency are relevant for also impacting viral production estimates. Most studies use 3‐h intervals over a 9 or 12 h period, with some adding an additional 24 h sample (Table S1). In (highly) productive coastal waters, subsampling every 1–2 h over a shorter total incubation time is preferred, whereas longer subsampling frequencies (every 6–7 h) and incubation duration (up to 70 h) have been reported for oligotrophic open ocean studies (Table S1). Moreover, high bacterial growth rate, short phage latent period and high burst size can contribute towards increased re‐infection, causing overestimation of lytic viral production (and underestimation of induced viral production) if not corrected for by shortening the incubation duration (Winget et al. 2005; Noble and Fuhrman 2000; Parada et al. 2007; Motegi et al. 2013). As the viral production assay aims at measuring viruses produced only from the infections that occurred prior to the assay, knowledge on how long to incubate will aid in accurately estimating viral production rates.

In this study, we performed a literature survey to assess the prevalence of linear regression and VIPCAL approaches for estimating lytic and mitomycin C‐induced viral production rates. We then provide evidence of differences in viral production rates estimated between linear regression and VIPCAL by simulating viral production assay datasets in silico, emphasising the need for further standardisation of methodologies when estimating viral‐mediated bacterial mortality and lysogeny. We introduce VIPCAL‐SE, where we improve VIPCAL by adding stricter maxima and minima scouting, ensuring estimations with higher confidence. We also highlight the importance of considering the duration of the incubation driven by bacterial growth in avoiding over‐ and underestimations in viral production calculations. Lastly, we compare the performance of VIPCAL‐SE with linear regression and VIPCAL in different marine environments to test the applicability of the approach in estimating viral production rates under different trophic statuses.

## 2 Results and Discussion

### 2.1 Comparing Prevalent Analytical Approaches

**FIGURE 2:**
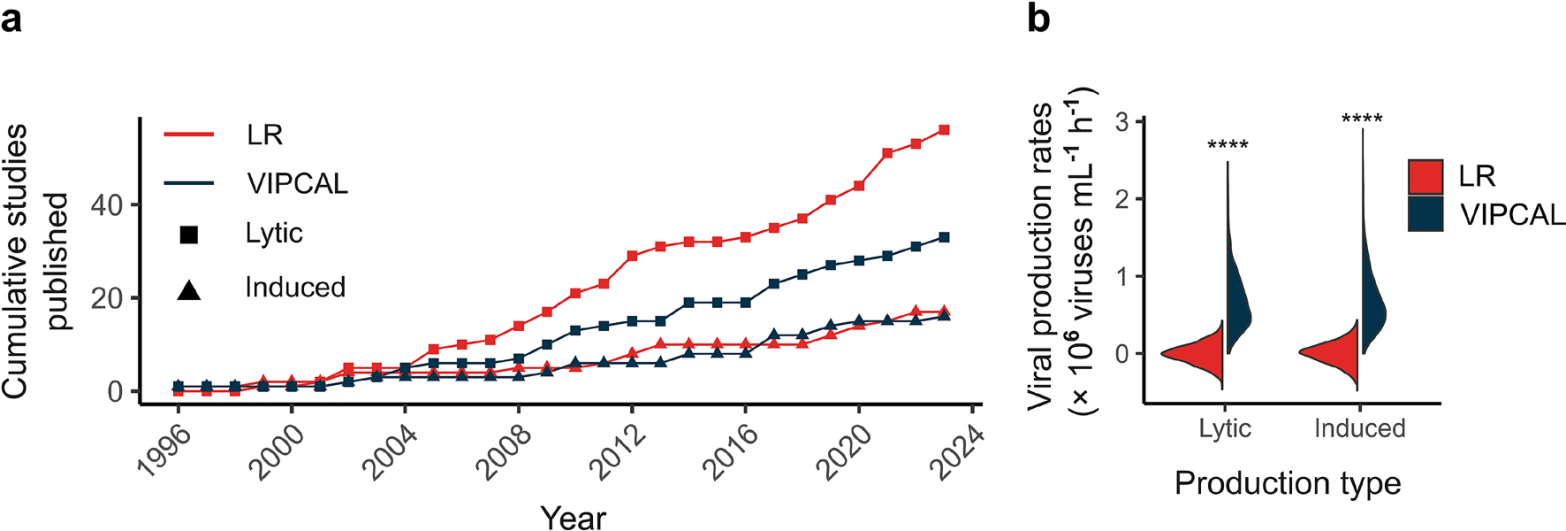
Comparison of data analysis approaches used for viral production rate estimations. (a) Cumulative number of studies published that estimated lytic and induced viral production rates using the linear regression and VIPCAL approaches from 1996 to 2024. (b) Distribution of lytic and induced viral production rates determined from the Monte Carlo simulation using the linear regression and VIPCAL approaches. Significant differences in the estimates are highlighted (****p < 0.0001).

Viral production assays have been the gold standard for estimating viral production rates in mixed aquatic bacterial communities for about three decades now (Xie et al. 2021; Winget et al. 2005). We identified a total of 89 studies that performed viral production assays to estimate lytic viral production rates due to viral‐mediated bacterial mortality, of which 33 studies also estimated the prevalence of lysogeny by adding the antibiotic mitomycin C as an inducing agent (Figure 2a). Of the studies compiled, 64% employed the linear regression approach to estimate lytic viral production rates, whereas both the linear regression and VIPCAL approaches were equally prevalent when calculating induced viral production rates.

Given the common use of the linear regression and VIPCAL approaches in estimating viral production rates, it is pertinent to understand how the estimates compare between these two approaches. However, as most viral production studies have not published the actual viral counts per replicate per time point, we cannot utilise existing studies to compare viral production rate estimates. Therefore, we performed a Monte Carlo simulation to generate 1000 viral production assay datasets (VP and VPC treatments) in silico that were subsequently used to assess the lytic and induced viral production rates extracted using the linear regression and VIPCAL approaches.

Existing studies that employed the linear regression approach differed slightly in the order in which they treated the data between replicates and time points. Some studies calculated individual slopes per replicate and then averaged them to obtain the overall average viral production rate (LR‐1), whereas others averaged viral counts per time point and then obtained the overall production rate from the single slope (LR‐2). Lastly, there were a few studies that computed a single slope by combining viral counts from all replicates without averaging viral counts (LR‐3). Despite these variations in the order of data handling, the calculated mean viral production rates did not differ between these approaches (Kruskal–Wallis rank sum test, chi‐squared = 1.8348e−05, *p* = 1) (Figure S1a). This is simply due to the linearity of summations. Standard errors (SE) calculated by these approaches, however, differed significantly (Kruskal–Wallis rank sum test, chi‐squared = 500.9, *p* < 0.0001) from each other (Figure S1b). Standard deviations (using Dunn's post hoc test with Bonferroni correction) were significantly lower for LR‐1 compared to LR‐2 (*Z* = −22.14, adjusted *p* < 0.0001) and LR‐3 (*Z* = −13.92, adjusted *p* < 0.0001), while LR‐2 has a significantly higher SE calculation than LR‐3 (*Z* = 8.22, adjusted *p* < 0.0001). As LR‐1 captures potential biological variability between replicates before averaging them, we deem LR‐1 (hereafter referenced as the ‘linear regression’ approach) the most appropriate representation for the linear regression approach.

We applied a fixed assay duration of six time points to compare lytic and induced viral production rates estimated using linear regression and VIPCAL approaches. Significantly higher lytic (Kruskal–Wallis rank sum test, chi‐squared = 1441.6, *p* < 0.0001) and induced (Kruskal–Wallis rank sum test, chi‐squared = 1417.7, *p* < 0.0001) viral production rates were observed when using VIPCAL as compared to linear regression (Figure 2b). The only other viral production study reporting viral production rates estimated using both approaches also found higher rates by VIPCAL as compared to linear regression (Ho et al. 2021). Considering the prevalence of both linear regression and VIPCAL in the literature and the significant differences in the viral production rates estimated by these approaches, caution is advised when comparing findings from studies employing different approaches. Similarly, meta‐analysis studies should consider the impact of the analytical method used, and public databases, such as the global Viral Oceanography Database (gVOD) (Xie et al. 2021), should report the analytical approach used for the viral production estimations.

### 2.2 Upgrading VIPCAL to VIPCAL‐SE

**FIGURE 3:**
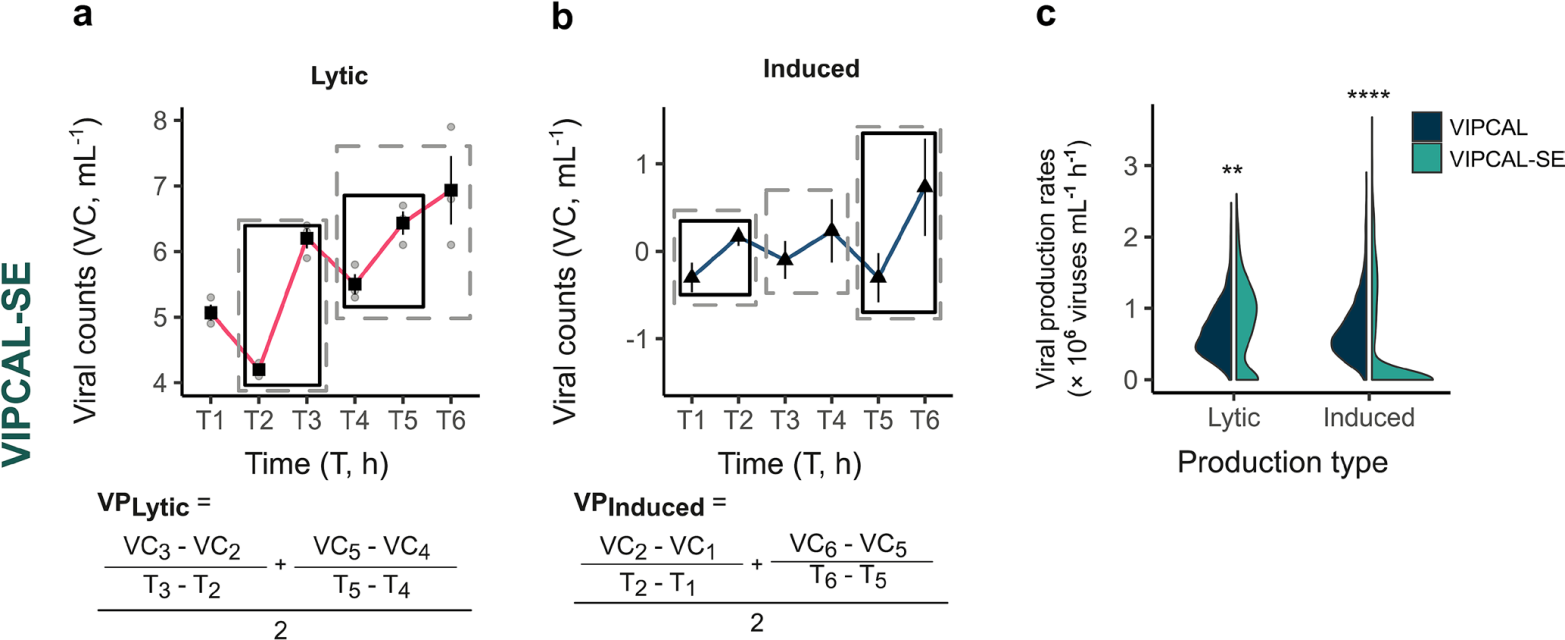
VIPCAL‐SE for viral production rate estimations. Addition of the SE of the means ensures high confidence maxima–minima pairs are utilised for lytic (a) and induced (b) viral production rate estimates. VIPCAL‐SE uses VIPCAL approach by analysing increments in viral counts (VC; in viruses mL^−1^) over time (*T*; in h) to estimate viral production rates. SE of the means are calculated on the difference curve by using the combined SE formula. Periods of productivity considered by VIPCAL and VIPCAL‐SE are highlighted using grey dashed boxes and black solid boxes, respectively. (c) Distribution of lytic and induced viral production rates determined using VIPCAL and VIPCAL‐SE approach, highlighting significant differences in the estimates (****p < 0.0001, **p < 0.05).

VIPCAL currently ignores spread in viral counts between replicates (as it averages the viral counts per time point per treatment), making it prone to erroneous identification of maximum–minimum pairs due to potential outliers. Statistical analysis techniques such as outlier analysis, *t*‐test and Mann–Whitney test are deemed inappropriate for maxima–minima scouting due to the typically small replicates group size (*n* = 2–5, typically *n* = 3). Therefore, we incorporated SE of the mean to assess true maxima and minima in our dataset. As per our model, a maximum–minimum pair is identified when the mean + SE of viral counts at time point *T*_*n*_ is smaller than the mean − SE at *T*_(*n* + 1)_, that is, their SE do not overlap (Figure 3a,b). To elaborate, we first identify maxima and minima pairs based on mean viral counts. The validity of each pair is then tested by ensuring non‐overlapping SE. To further refine the period of productivity, each minimum is compared with the next timepoint to ensure a significant difference. If the SE overlap, the minimum is iteratively shifted to the next time point until a significant difference is observed or the next time point corresponds to a maximum. We refer to this refined model as ‘VIPCAL‐SE’.

Comparing VIPCAL‐SE with VIPCAL, it estimates ‘zero’ or no lytic viral production rates for 31% of the simulated dataset, whereas VIPCAL estimates it for 0.1% of all cases. Furthermore, lytic viral production estimates differ significantly (Figure 3c; Kruskal–Wallis rank sum test, chi‐squared = 9.07, *p* < 0.05), with higher rates estimated by VIPCAL‐SE in nearly half of the simulated datasets. Generally, stricter maxima–minima identifications by VIPCAL‐SE lead to higher production estimations due to the rates being calculated over a shorter period. To summarise, VIPCAL‐SE reduces overall estimations of lytic viral production rates but provides higher production rates than VIPCAL and with greater confidence due to the incorporation of SE of means in identifying maximum–minimum pairs.

When extracting lysogeny occurrence, we calculated the SE of the difference curve between VPC and VP using two approaches, that is, a combined SE formula (Equation 1, Methods) and a linear mixed effect regression model. The latter model explains variations in viral counts with treatment and time point, along with their interactions, as fixed effects, and a random effect for replicates to account for variability between them. Significant differences were observed in viral production due to lysogenic inductions between VIPCAL‐SE modified with SE using the combined SE formula and the linear mixed effect regression model (Figure S2; Kruskal–Wallis rank sum test, chi‐squared = 19.152, *p* < 0.0001). With a maximum of 18 data points (three replicates, six time points), using a linear mixed effect regression model to precisely estimate the SE of the difference curve may lead to overfitting, which could cause further analytical errors. Therefore, we conclude that the combined SE formula is the most appropriate method to calculate SE the difference curve.

The induced viral production rates obtained using VIPCAL‐SE were significantly lower than those retrieved using VIPCAL (Figure 3c; Kruskal–Wallis rank sum test, chi‐squared = 496.43, *p* < 0.0001). VIPCAL provided higher estimations on 77% of the simulated dataset. VIPCAL‐SE estimated ‘zero’ or no induced viral production for 76% of the dataset, compared to only 0.3% of VIPCAL. The inclusion of SE of means in VIPCAL‐SE thus reduces the incidence of potentially false induced viral production estimations that could have been propagated by erroneous identification of maximum–minimum pairs due to outliers in viral counts. VIPCAL‐SE provided higher lytic and induced viral production rates than linear regression as it only focuses on periods of productivity (Figure S3).

### 2.3 Determining Assay Endpoint

**FIGURE 4:**
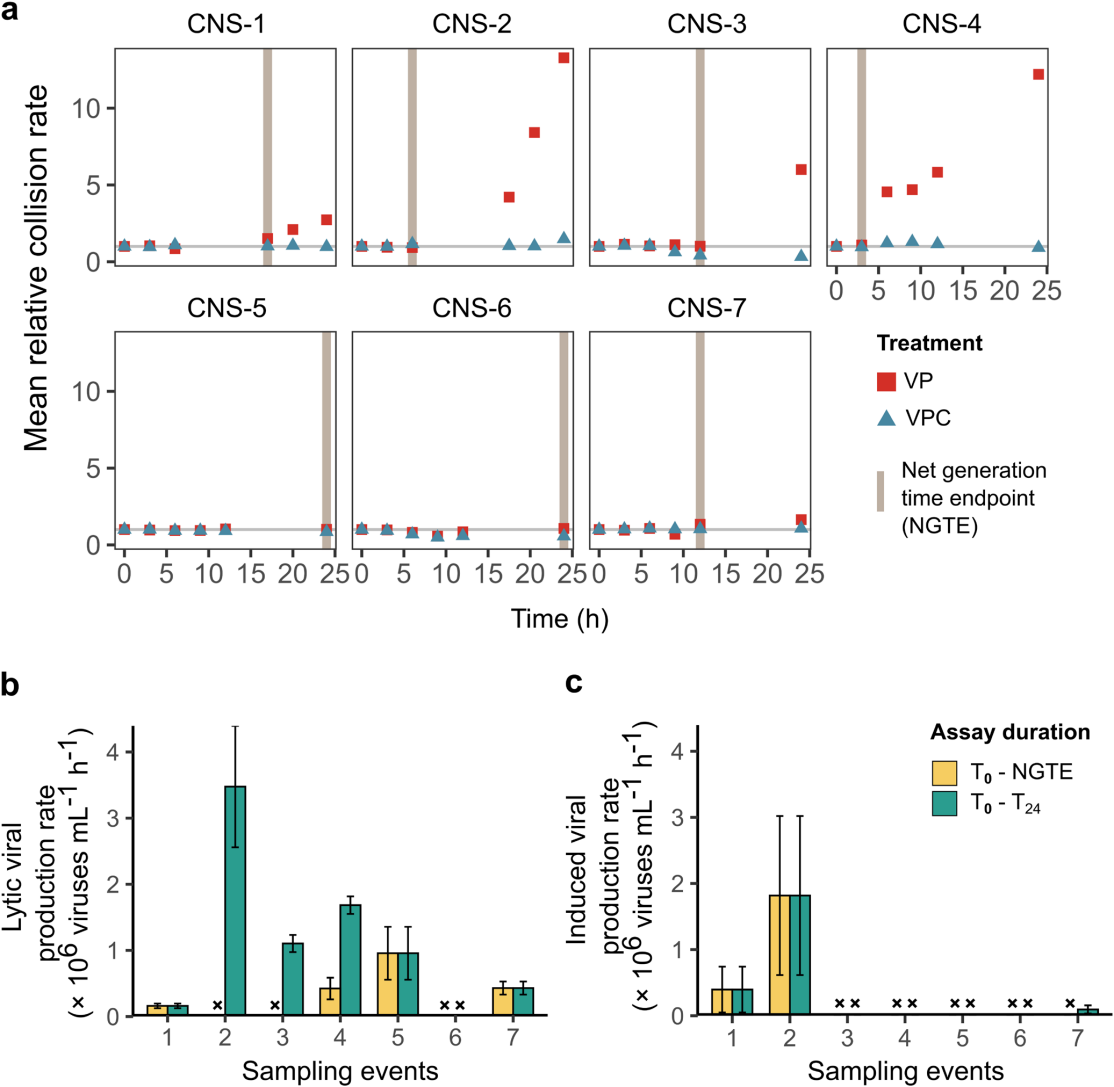
Impact of incorporating bacterial net generation time endpoint (NGTE) in viral production calculations for seven viral production assays performed at the coastal Dutch North Sea. (a) Evolution of mean relative collision rate between bacteria and viruses in VP (no mitomycin C addition) and VPC (mitomycin C) treatments over time. A cutoff time point (NGTE) was determined for each assay, where it is the timepoint at which net bacterial generation time in the VP treatment is less than 24 h. This minimises the chance of secondary infections and release of viruses during the incubation. A divergence in mean relative collision rates were observed between the two treatments after NGTE. Comparison of lytic (b) and induced (c) viral production rates estimated using VIPCAL‐SE at NGTE (*T*_0_ to NGTE) and for the entire duration of the assay (*T*_0_–*T*_24_). The symbol ‘×’ denotes instances where no detectable viral production was observed.

One of the key principles of the viral production assay is to measure viral production exclusively from infections that happened before the start of the assay. Overestimations potentially occur if new productive infections occur during the assay. The viral production assay is designed to reduce the collision rate between bacteria and viruses by washing out viruses prior to incubation to minimise new infections. However, if the bacterial production is high (leading to higher standing stock) and viral proliferation is high, the collision rate between the bacterial host and viruses increases. Consequently, the risk of secondary infections later in the assay increases, leading to overestimations in viral production rates (Winget et al. 2005; Motegi et al. 2013). Bacterial production and growth vary depending on sampling location, season, time of the day, depth, photosynthetically active radiation intensity, nutrient availability, among other factors. To prevent the release of virus progeny from secondary infections, it is relevant to consider the duration of the viral production assay (Noble and Fuhrman 2000). Ideally, the bacterial gross growth rate is assessed for each assay prior to incubation to determine maximum incubation time. This is, however, most often practically not feasible during scientific cruises. Besides, viral production assays are generally performed without additional bacterial production measurements. Therefore, we recommend an extended incubation period of 12 h (or even 24 h) and increased subsampling frequency, whereby the actual duration is determined after sample processing based on bacterial net growth rate (from flow cytometric bacterial enumeration on samples used for viral counting). For each assay, we defined a cutoff time as the last sampling point before the net bacterial generation time (Equations 2 and 3)—calculated relative to the 0 h subsample—in the unamended (VP) treatment exceeded 24 h. This minimises the chance of secondary infections and release of viruses during the incubation. We call this time point the net generation time endpoint (NGTE). We performed seven viral production assays in the coastal North Sea (CNS) waters of the Netherlands, and a large variation in bacterial growth was already observed during the 2 months sampling period. Bacterial net growth rates varied from −0.002 to 0.03 d^−1^, resulting in NGTEs of 3–24 h, demonstrating the relevance of taking incubation duration into consideration, especially in highly productive coastal waters (Figure 4a).

We determined the relative virus‐bacterial host cell collision rate (Equation 4, Methods) compared to the start of the incubation (*T*_0_) to assess whether net bacterial growth (expressed as NGTE) serves as a reliable indicator for incubation duration. In the VP treatments, collision rates at the end of the 24 h incubations (Figure 4a) ranged from 0.95 to 16.9 (mean ± standard deviation, 5.34 ± 5.27), relative to the initial rate at *T*_0_. The temporal dynamics show that the collision rate did not increase substantially before the calculated NGTE, supporting the use of NGTE to estimate the maximum duration of incubation for the viral production assay. Still, collision rates clearly increased after the NGTE in four of the seven assays, demonstrating that adaptation of the duration length is indeed recommended. We calculated lytic viral production using VIPCAL‐SE for the entire 24 h duration and until the specific NGTE. We observed that lytic viral production rates calculated at NGTE were significantly lower than at *T*_24_ (Figure 4b; Wilcoxon rank sum test, *W* = 2.5, *p* < 0.05). For samples 2 and 3, the lytic viral production rates at NGTE no longer showed viral production (Figure 4b). This test shows the potential for overestimating lytic viral production rates (due to secondary infections) when performing long incubations. The strong variation in these seven assays most likely represents fluctuations in microbial community structure as observed in dynamic coastal ecosystems that are also influenced by tides, riverine freshwater input and terrestrial runoffs (Aguilar et al. 2025; Doan et al. 2014; Marín‐Vindas et al. 2023; Meyneng et al. 2024; Van Duyl and Kop 1988).

As expected, the VPC treatment did not show increases in relative collision rates (Figure 4a; mean ± standard deviation, 0.88 ± 0.36). The antibiotic mitomycin C has an inhibitory effect on bacterial growth due to DNA cross‐linking, DNA alkylation and production of reactive oxygen species in bacterial hosts (Tomasz 1976, 1995; Dapa et al. 2017). A strong difference between bacterial net growth rates between the VPC and the VP treatments complicates the extraction of induced viral production rates from the VPC treatment. We observed, however, that at NGTE the relative collision rates between the VP and VPC treatments remained comparable (Figure 4a), further strengthening the application of NGTE for final virus production rate extraction. We applied the VIPCAL‐SE approach to determine induced viral production rates for the entire assay duration (*T*_0_–*T*_24_) and until the NGTE. Mitomycin C‐based inductions were observed in three out of seven assays using VIPCAL and in two using VIPCAL‐SE, with no significant differences found between *T*_0_–*T*_24_ and *T*_0_–NGTE induced viral production rate calculations (Figure 4c; Wilcoxon rank sum test, *W* = 11, *p* > 0.05).

### 2.4 Comparing Estimations Across Seas

**FIGURE 5:**
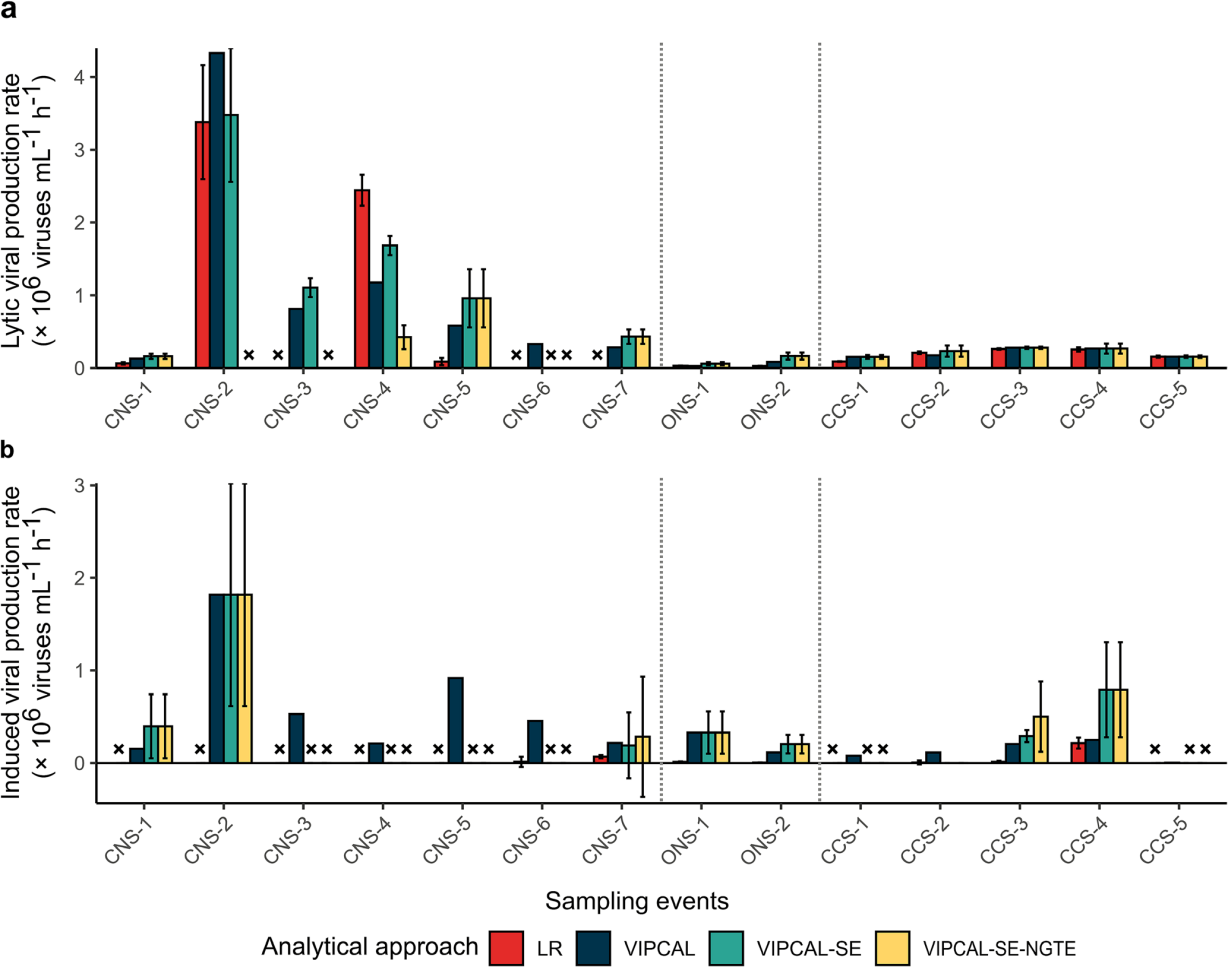
Viral production estimates calculated using four analytical approaches: Linear regression, VIPCAL, VIPCAL‐SE and VIPCAL‐SE at the net generation time endpoint (NGTE) in three marine environments—coastal North Sea (CNS), open North Sea (ONS) and the Curaçao Caribbean Sea (CCS). The NGTE varied by sample: *T*_3_ for CNS‐4; *T*_6_ for CNS‐2; *T*_12_ for CNS‐3, CNS‐7 and CCS‐3; *T*_17_ for CNS‐1 and *T*_24_ for all other samples. The symbol ‘×’ denotes instances where no detectable viral production was observed. All data can also be found in Table S2.

We compared the performance of VIPCAL‐SE with linear regression and VIPCAL in estimating viral production rates across three marine environments (Figure 5): (i) the temperate, CNS around the Netherlands, which was mesotrophic at the time of sampling (*n* = 7); (ii) the oligotrophic, open North Sea (ONS) in summer (*n* = 2); and (iii) the oligotrophic, tropical, coastal waters of Curaçao in the Caribbean Sea (CCS; *n* = 5). We also incorporated the NGTE as a factor for VIPCAL‐SE that has not explicitly been considered in determining viral production rates in the linear regression and VIPCAL approaches previously. In contrast to the CNS samples presented earlier (with high variation in bacterial net generation time, resulting in a wide range of NGTEs), the net generation times in the ONS and the CCS samples were consistently > 24 h (NGTE is *T*_24_) with the exception of CCS‐3, where the endpoint was determined to be *T*_12_ (Figure S4). This screening indicates that agreement between the analytical approaches estimating viral production depends on the trophic state of the sampled waters (Figure 5a).

All approaches in the oligotrophic Caribbean Sea provided similar estimates, most likely due to relatively low bacterial net growth, steady virus production and no explicit viral loss (Figure S4). In the open North Sea, the VIPCAL‐SE estimates were higher, not due to NGTE adjustment but most likely because of the relatively high SE leading to shortening of the considered period of increase. Similarly, the Dutch coastal water station CNS‐5 showed higher lytic viral production rates with VIPCAL‐SE. Our employment of the NGTE as an in situ control to determine the maximum incubation period lead to reduction in estimates for Dutch coastal water stations (CNS‐2–4). We observed higher discrepancies between the different approaches in estimating the induced viral production, irrespective of the trophic state of the sampled water (Figure 5b). While VIPCAL estimated induced viral production for all 14 samples, VIPCAL‐SE estimated it for only half. By incorporating SE, VIPCAL‐SE reduced overestimations by VIPCAL. Additionally, VIPCAL‐SE provided higher estimates than linear regression for five stations (CNS‐7, ONS‐1, ONS‐2, CCS‐3 and CCS‐4), as it only focused on periods of productivity. In summary, incorporation of SE provided viral production estimates with higher confidence across trophic status, while NGTE reduced potential overestimation due to high bacterial growth in productive coastal seas assays (that could potentially lead to secondary viral production if not corrected for).

Negative values may occur during viral production analyses (Knowles et al. 2017). In the linear regression approach, induced viral production is calculated by subtracting the VP slope from the VPC slope. This can result in a negative rate if the slope of the VPC treatment is lower than that of VP (CNS‐1‐5, CCS‐1, CCS‐5). A negative difference curve is also possible when applying VIPCAL or VIPCAL‐SE. However, as these approaches focus on increments, the induced viral production estimated will be positive or zero at worst. Our study shows that a possible cause is the overestimation of lytic viral production due to high bacterial growth, leading to underestimation of lysogeny by the linear regression approach and consequently producing negative viral production rates (CNS‐2). Alternatively, mitomycin C based inductions may not fully represent community‐wide lysogeny, as they selectively induce only certain fractions of lysogens and are also dose‐dependent (Weinbauer and Suttle 1996; Sekiguchi and Takagi 1960; Knowles et al. 2017; Ackermann and DuBow 1987; Paul 2008). While selective induction should theoretically lower induced production estimates, the presence of negative induced viral production still suggests that mitomycin C might affect lytic viral production as well (potentially by killing bacterial cells before viral mediated cell lysis or not allowing progeny viruses to be released). Viral production assays traditionally found no impact of mitomycin C on viral DNA production in T2r‐, T3‐ and T5‐infected *Escherichia coli* (Sekiguchi and Takagi 1960). The effect of mitomycin C on lytic production in mixed marine bacterial communities is still largely unclear and warrants further investigation to refine lysogeny estimations.

### 2.5 Considerations in Further Refining Viral Production Estimates

While this study focuses on extracting more accurate viral production rates from the viral counts in the viral production assay, we recommend assessing several factors before and after rate extractions. The subsampling frequency and total incubation period are important factors to be considered when performing a viral production assay. Our study shows that it is well justified to adapt a higher subsampling frequency in more productive (coastal) waters, as compared to more oligotrophic waters such as the open ocean (Table S1). We recommend hourly or even half‐hourly subsampling for the first 6 h of incubation. In contrast, for less productive waters, we recommend incubations up to 24 h to ensure production of viruses with long latent periods and/or delayed production upon induction with mitomycin C are also accounted for. To be safe, the assay duration can be longer in practice as it can be estimated post hoc by the principles of NGTE. Another source of uncertainty lies in the estimation of viral burst size, which is essential to translate viral production rates to viral‐mediated bacterial loss, carbon and nutrient release, and the prevalence of lysogeny. A community burst size is often assumed from literature values, typically based on previous studies at similar locations or conditions. However, burst sizes may vary for the different bacterial host groups (Parada et al. 2006) and are dependent on the metabolic state of the bacterial host, type of virus, time of infection and host composition. Ideally, burst size must be calculated for every community (e.g., using transmission electron microscopy; Parada et al. 2006; Weinbauer et al. 2002; Shruthi et al. 2022), but this is generally not feasible for every laboratory. We therefore recommend that authors of future studies provide not only the assumed burst size but also the raw microbial counts and production rates from their studies to facilitate future meta‐analyses.

Lastly, a 0.22 μm filtered control treatment is commonly included to check for potential loss of viruses by adsorption to the walls of the incubation tubes or to small‐sized colloidal particles (Maat, Visser, et al. 2019; Maat, Prins, et al. 2019; Richter et al. 2021). Note that corrections for viral loss must be applied per sampling point before viral production rates are estimated. Our study showed high viral loss in the coastal waters with high turbidity. Given that turbidity and colloid particle composition vary across environments (Shi and Wang 2010), an improved understanding of the underlying loss mechanisms is recommended.

## 3 Conclusions

Accurate and standardised estimations of viral‐mediated bacterial mortality and prevalence of lysogeny are important factors when deciphering the ecological role of viruses in any given environment. We provided a comprehensive assessment of the two major existing analytical approaches (linear regression and VIPCAL) in extracting lytic and induced viral production rates from the viral production assays. While linear regression considers biological variability between replicates when determining production rates, it is more subjective as it relies on expert evaluation to determine periods of productivity. VIPCAL has an edge as it accounts for fluctuations in viral counts and is standardised as an online tool, enhancing reproducibility, but is also susceptible to overestimations due to variability between replicates and potential outliers. We introduced VIPCAL‐SE, where we improved on VIPCAL by incorporating SE of means when identifying maximum–minimum pairs. This accounts for biological and ecological variability in viral counts overlooked by VIPCAL. We also introduced a bacterial NGTE cut off to more precisely define the assay duration as an in situ control, particularly in coastal and estuarine regions with rapid spatiotemporal fluctuations in bacterial net growth rates. We demonstrated that VIPCAL‐SE improves the quality of the lytic and induced viral production rates as compared to linear regression and VIPCAL. While VIPCAL‐SE through the *viralprod* R package delivers increased reproducibility and standardisation, we want to highlight the importance of visual inspection of the dynamics of viral counts over the assay duration to determine any potential outliers that might interfere with viral production estimations using VIPCAL‐SE. With thorough inspection of viral counts from the viral production assay, incorporation of SE of means and employment of NGTE, VIPCAL‐SE minimises errors in viral production rate calculations, aiding in more accurate estimations of bacterial mortality and lysogeny, especially in highly productive environments.

## 4 Methods

### 4.1 Literature Survey

A non‐exhaustive, approach‐centric literature search was performed to identify viral production studies employing viral production assays. We surveyed the Web of Science Core Collection on 28 May 2024, using the search string in the topic field ‘(TS = (marine AND (virus* OR viral) AND (bacteri* OR microb* OR prokaryot*) AND (production OR mortality OR lysis)))’ to extract studies concerning viral production or bacterial mortality in the marine environment. We only retained studies that used one of the three following virus reduction approaches—dilution with virus‐free water, viral reduction over a 0.22 μm membrane filter/vacuum pump setup, and tangential flow filtration for comparison. Studies on lysogeny that did not use mitomycin C to induce lysogens were excluded. Information on the year of publishing, type of virus reduction approach employed, number of assays performed for lytic and induced viral production estimations, subsampling time points and the data analysis methods used (linear regression model‐based or increment‐based (VIPCAL) approaches) were extracted (Table S1).

### 4.2 Simulation Dataset Generation

As most viral production studies have not published the direct viral counts per replicate per time point, we cannot utilise published studies to compare the performance of the linear regression model with VIPCAL in extracting viral production rates. To circumvent this, we used a Monte Carlo approach to simulate a dataset of 1000 viral production assays in silico. Each assay consisted of two treatments (VP for lytic viral production and VPC for lytic and induced viral production), three replicates per treatment and six subsampling time points (*T*_1_–*T*_6_). A mean viral count was randomly assigned per treatment per time point with values ranging between and including two and three. To further incorporate noise between replicates due to biological and technical reasons, we randomly assigned a percentage standard deviation (% SD) between 0% and 100%. Standard deviation (SD) was calculated by multiplying % SD with mean viral counts. We used the mean viral count and SD to generate three random positive numbers that were assigned to each replicate, per treatment, per time point. This dataset was used to assess viral production rates.

### 4.3 Viral Production Assay

To understand the implication of assay duration, we performed seven viral production assays in the Dutch CNS (August and September 2020), two in the open North Sea (ONS; September 2020 and April 2021) and five in the Caribbean Sea around Curaçao (CCS; April 2022). These assays followed the approach of Winget et al. (2005), with a modification: surface seawater samples were gently (reversely) pre‐sieved through a large 50 μm pore size sieve and siphoned into a polycarbonate carboy to exclude phytoplankton colonies (e.g., *Phaeocystis globosa* late summer blooms) and larger organisms, without lysing bacterial cells, to avoid clogging the filtration cartridge. Briefly, virus‐free seawater was generated using a 30 kDa polyether sulfone (PES) membrane Vivaflow 50 tangential filtration flow (TFF) cartridge (Sartorius, Germany). To reduce the viral abundance, 300 mL of seawater sample was washed with virus‐free seawater using a 0.22 μm PES Vivaflow 50 TFF cartridge (Sartorius, Germany) at a permeate discharge rate of 40 mL min^−1^. When the retentate volume was reduced to 50 mL, 250 mL of virus‐free seawater was added and allowed to reduce back to 50 mL. This step was repeated two more times or until a total of 750 mL of virus‐free seawater was used for flushing. The 50 mL retentate was topped with 250 mL of virus‐free seawater and aliquoted into six 50 mL Greiner tubes (Sigma‐Aldrich, USA). Three tubes were left unamended to quantify lytic viral production (VP treatment), whereas the antibiotic mitomycin C (final concentration, 1 μg mL^−1^, Sigma‐Aldrich, USA) was added to the other three tubes to induce prophages into entering the lytic cycle (VPC treatment). Additionally, 0.22 μm filtered seawater was incubated in parallel in triplicate to assess viral loss due to adsorption to Greiner tube walls. Sample tubes were incubated in the dark in a water bath at the in situ temperature. After gentle mixing, 1 mL aliquots were taken at 0, 3, 6, 9, 12 and 24 h (for CNS‐1 and CNS‐2, at 0, 3, 6, 17, 20 and 24 h), fixed with 0.5% glutaraldehyde (25%, EM‐grade, Merck, USA) for 15 min at 4°C, flash frozen in liquid nitrogen, and stored at −80°C. All seawater processing was performed at in situ temperatures and dimmed light conditions.

### 4.4 Microbial Abundances

Bacterial and viral abundances were enumerated using SYBR Green I‐assisted flow cytometry on a Becton‐Dickinson FACSCalibur flow cytometer as outlined by Marie et al. (1999) and Brussaard (2004), with modifications by Mojica et al. (2014). In short, samples were fixed in 25% EM‐grade glutaraldehyde (final concentration, 0.5%; Sigma‐Aldrich, USA) for 30 min at 4°C, followed by flash freezing and storage at −80°C. For flow cytometric analysis, samples were thawed and diluted in Tris–EDTA buffer (pH 8.2, 10 mM Tris–HCl, 1 mM EDTA; Sigma‐Aldrich, USA) filtered with FP 30 mm 0.22 μm syringe filter (Whatman Puradisc, Sigma‐Aldrich, USA) to obtain an event rate of 200–800 events s^−1^. The samples were stained with the nucleic acid‐specific dye SYBR Green I (final concentration, 1.0 × 10^−4^ of the commercial stock, Thermo Fisher Scientific, USA) and incubated in the dark for 10 min at 80°C for viral enumeration, after which they were cooled down for at least 5 min before analysis. For bacterial enumeration, samples were stained for 10 min in the dark at room temperature. The stained samples were counted based on green fluorescence versus side scatter upon excitation with a 488 nm Argon‐Ion (15 mW) laser.

### 4.5 Analyses

All data handling was done in R Statistical Software (Team, R. C 2000) in RStudio (Racine 2012) using the *tidyverse* R package (Wickham et al. 2019). Raw flowcytometry data files were processed using the *flowCore* (Hahne et al. 2009) and *flowWorkspace* (Finak et al. 2012) R packages to extract bacterial and viral counts for viral production rate estimations. Viral loss rates were calculated as the slope of a first‐order linear regression fitted to viral abundance over time in the 0.22 μm incubations. The production rates were estimated on viral loss corrected viral counts by first‐order linear regression and by VIPCAL (original and with modifications as described) using the *viralprod* R package (github.com/mdhishamshaikh/ViralProduction_R) (Shaikh et al. 2023). The combined SE of means of the difference curve for the VIPCAL‐SE approach was calculated using Equation (1):

(1) $$\text{Difference Curve Standard Error}=\sqrt{{\mathrm{SE}}_{\mathrm{VPC}}^{2}+{\mathrm{SE}}_{\mathrm{VP}}^{2}-2\mathrm{Cov}\left({\mathrm{MVC}}_{\mathrm{VPC}},{\mathrm{MVC}}_{\mathrm{VP}}\right)\;}$$

where SE is the standard error of means and MVC is the mean viral counts at given time point.

The net bacterial growth rate at any given subsampling time was calculated using Equation (2):

(2) $$\mu =\frac{\ln{\mathrm{BA}}_{T_{n}}-\ln{\mathrm{BA}}_{T_{0}}}{T_{n}-T_{0}}$$

where *μ* is the bacterial growth rate and BA is the bacterial abundance at a given time *T*.

The net bacterial generation time was calculated using Equation (3):

(3) $$\mathrm{GT}=\frac{\ln2}{\mu }$$

where GT is the generation time and *μ* is the bacterial growth rate.

Relative collision rates were calculated with respect to *T*_0_ using Equation (4):

(4) $$\text{Relative Change in collision rate}\;\mathrm{at}\;T_{n}=\frac{{\mathrm{BA}}_{T_{n}}\times {\mathrm{VA}}_{T_{n}}}{{\mathrm{BA}}_{T_{0}}\times {\mathrm{VA}}_{T_{0}}}$$

where BA and VA are bacterial and viral abundance at a given time *T*, respectively.

Viral production rates were compared between groups using the Kruskal–Wallis and Wilcoxon rank sum tests from the *stats* R package. Additionally, Dunn's test from the *dunn.test* R package (Dinno and Dinno 2017) was applied as a post hoc analysis to control for multiple comparisons and to identify specific group pairs with significant differences. Plots were generated using the *ggplot2* R package (part of *tidyverse*) and compiled using Inkscape (Inkscape Project 2024).

## Author Contributions

**Hisham M. Shaikh:** conceptualization, methodology, software, visualization, investigation, writing – original draft, writing – review and editing, validation, formal analysis, data curation. **Jonas Van den Bremt:** software; writing – review and editing. **Lisa Schellenberg:** investigation; writing – review and editing. **Salvador J. Fernández Bejarano:** software; writing – review and editing. **Maarten De Rijcke:** conceptualization; methodology; writing – review and editing. **Corina P. D. Brussaard:** conceptualization; methodology; writing – review and editing.

## Funding

This work was supported by Vlaams Instituut voor de Zee; Nederlandse Organisatie voor Wetenschappelijk Onderzoek (NWOCA.2019.003) and Fonds Wetenschappelijk Onderzoek (I002021N).

## Conflicts of Interest

The authors declare no conflicts of interest.

## Supporting information


**Figure S1:** Comparison of three variations in the linear regression model (LR) analysis approach in extracting viral production rates. (a) No significant differences were observed in lytic and induced viral production estimates between the three variants. (b) Significant differences (*p* < 0.0001) were observed in the standard errors calculated between the three LR variants used to estimate both lytic and induced viral production rates.
**Figure S2:** Impact of using combined standard error formula (Equation 1) and applying a linear mixed effects model (LMER) to extract standard errors of the difference curve when estimating mitomycin C‐induced viral production rates using VIPCAL‐SE approach. Significant differences were observed between the two approaches (****p < 0.0001).
**Figure S3:** Comparison of lytic and induced viral production rates estimated using LR, VIPCAL and VIPCAL‐SE approaches. All approaches provide significantly different estimates from each other (*p* < 0.0001).
**Figure S4:** Evolution of mean relative collision rate between bacteria and viruses in VP (no mitomycin C addition) and VPC (mitomycin C) treatments over time in viral production assays performed in coastal North Sea (CNS), open North Sea (ONS) and Curaçao Caribbean Sea (CCS). Net generation time endpoint (NGTE) is the timepoint at which net bacterial generation time in the VP treatment is less than 24 h. A divergence in mean relative collision rates were observed between the two treatments after NGTE.

**Table S1:** A literature survey of viral production studies using linear regression and increment‐based (VIPCAL) approaches.

**Table S2:** Metadata and results of viral production performed in the open North Sea, coastal North Sea and coastal waters of Curaçao in the Caribbean Sea.

## Data Availability

The data and analysis scripts for this study are available in a public repository on GitHub at https://github.com/mdhishamshaikh/Viral_Production_Method_Comparison. The *viralprod* R package used in this study is archived on Zenodo and is accessible via its DOI: https://doi.org/10.5281/zenodo.14945312 (development version available at https://github.com/mdhishamshaikh/ViralProduction_R/tree/rpkg).

## References

[emi470258-bib-0001] Ackermann, H. W. , and M. S. DuBow . 1987. “Description and Identification of New Phages.” In Viruses Prokaryotes, vol. 1, 103–142. CRC Press Inc.

[emi470258-bib-0002] Aguilar, P. , C. Piyapong , N. Chamroensaksri , P. Jintasaeranee , and R. Sommaruga . 2025. “Tidal Levels Significantly Change Bacterial Community Composition in a Tropical Estuary During the Dry Season.” Marine Life Science & Technology 7, no. 1: 144–156. 10.1007/s42995-024-00254-w. 40027330 PMC11871172

[emi470258-bib-0003] Bar‐On, Y. M. , R. Phillips , and R. Milo . 2018. “The Biomass Distribution on Earth.” Proceedings of the National Academy of Sciences 115: 6506–6511. 10.1073/pnas.1711842115 PMC6016768 29784790

[emi470258-bib-0004] Brussaard, C. P. D. 2004. “Optimization of Procedures for Counting Viruses by Flow Cytometry.” Applied and Environmental Microbiology 70: 1506–1513. 15006772 10.1128/AEM.70.3.1506-1513.2004 PMC368280

[emi470258-bib-0005] Brussaard, C. P. D. , X. Mari , J. D. L. V. Bleijswijk , and M. J. W. Veldhuis . 2005. “A Mesocosm Study of *Phaeocystis globosa* (Prymnesiophyceae) Population Dynamics.” Harmful Algae 4: 875–893.

[emi470258-bib-0006] Chu, T.‐C. , S. R. Murray , S.‐F. Hsu , Q. Vega , and L. H. Lee . 2011. “Temperature‐Induced Activation of Freshwater Cyanophage AS‐1 Prophage.” Acta Histochemica 113: 294–299. 20138651 10.1016/j.acthis.2009.11.003 PMC2891208

[emi470258-bib-0007] Cochran, P. , C. Kellogg , and J. Paul . 1998. “Prophage Induction of Indigenous Marine Lysogenic Bacteria by Environmental Pollutants.” Marine Ecology Progress Series 164: 125–133.

[emi470258-bib-0008] Danovaro, R. , C. Corinaldesi , A. Dell'Anno , et al. 2011. “Marine Viruses and Global Climate Change.” FEMS Microbiology Reviews 35: 993–1034. 21204862 10.1111/j.1574-6976.2010.00258.x

[emi470258-bib-0009] Dapa, T. , S. Fleurier , M.‐F. Bredeche , and I. Matic . 2017. “The SOS and RpoS Regulons Contribute to Bacterial Cell Robustness to Genotoxic Stress by Synergistically Regulating DNA Polymerase Pol II.” Genetics 206: 1349–1360. 28468910 10.1534/genetics.116.199471 PMC5500135

[emi470258-bib-0010] Dinno, A. , and M. A. Dinno . 2017. “Package ‘dunn.test’.” CRAN Repository 10: 1–7.

[emi470258-bib-0011] Doan, T. T. , C. Bouvier , Y. Bettarel , et al. 2014. “Influence of Buffalo Manure, Compost, Vermicompost and Biochar Amendments on Bacterial and Viral Communities in Soil and Adjacent Aquatic Systems.” Applied Soil Ecology 73: 78–86.

[emi470258-bib-0012] Evans, C. , and C. P. D. Brussaard . 2012. “Regional Variation in Lytic and Lysogenic Viral Infection in the Southern Ocean and Its Contribution to Biogeochemical Cycling.” Applied and Environmental Microbiology 78: 6741–6748. 22798377 10.1128/AEM.01388-12 PMC3426681

[emi470258-bib-0013] Evans, C. , J. Brandsma , M. P. Meredith , et al. 2021. “Shift From Carbon Flow Through the Microbial Loop to the Viral Shunt in Coastal Antarctic Waters During Austral Summer.” Microorganisms 9: 460. 33672195 10.3390/microorganisms9020460 PMC7927135

[emi470258-bib-0014] Evans, C. , I. Pearce , and C. P. D. Brussaard . 2009. “Viral‐Mediated Lysis of Microbes and Carbon Release in the Sub‐Antarctic and Polar Frontal Zones of the Australian Southern Ocean.” Environmental Microbiology 11: 2924–2934. 19758350 10.1111/j.1462-2920.2009.02050.x

[emi470258-bib-0015] Falkowski, P. G. , T. Fenchel , and E. F. Delong . 2008. “The Microbial Engines That Drive Earth's Biogeochemical Cycles.” Science 320: 1034–1039. 18497287 10.1126/science.1153213

[emi470258-bib-0017] Finak, G. , W. Jiang , J. Pardo , A. Asare , and R. Gottardo . 2012. “QUAliFiER: An Automated Pipeline for Quality Assessment of Gated Flow Cytometry Data.” BMC Bioinformatics 13, no. 1: 252. 23020243 10.1186/1471-2105-13-252 PMC3499158

[emi470258-bib-0016] Fortier, L.‐C. , and O. Sekulovic . 2013. “Importance of Prophages to Evolution and Virulence of Bacterial Pathogens.” Virulence 4: 354–365. 23611873 10.4161/viru.24498 PMC3714127

[emi470258-bib-0018] Hahne, F. , N. LeMeur , R. R. Brinkman , et al. 2009. “flowCore: A Bioconductor Package for High Throughput Flow Cytometry.” BMC Bioinformatics 10: 106. 19358741 10.1186/1471-2105-10-106 PMC2684747

[emi470258-bib-0019] Henrot, C. , and M. Petit . 2022. “Signals Triggering Prophage Induction in the Gut Microbiota.” Molecular Microbiology 118: 494–502. 36164818 10.1111/mmi.14983 PMC9827884

[emi470258-bib-0020] Ho, P.‐C. , G.‐C. Gong , C.‐H. Hsieh , P. W.‐Y. Chen , and A.‐Y. Tsai . 2021. “Diel Variation of Viral Production in a Coastal Subtropical Marine System.” Diversity 13: 426.

[emi470258-bib-0021] Howard‐Varona, C. , K. R. Hargreaves , S. T. Abedon , and M. B. Sullivan . 2017. “Lysogeny in Nature: Mechanisms, Impact and Ecology of Temperate Phages.” ISME Journal 11: 1511–1520. 28291233 10.1038/ismej.2017.16 PMC5520141

[emi470258-bib-0022] Inkscape Project . 2024. Inkscape. Inkscape Project. https://inkscape.org/.

[emi470258-bib-0023] Jiang, S. C. , and J. H. Paul . 1998. “Significance of Lysogeny in the Marine Environment: Studies With Isolates and a Model of Lysogenic Phage Production.” Microbial Ecology 35: 235–243. 9569281 10.1007/s002489900079

[emi470258-bib-0024] Knowles, B. , B. Bailey , L. Boling , et al. 2017. “Variability and Host Density Independence in Inductions‐Based Estimates of Environmental Lysogeny.” Nature Microbiology 2: 17064. 10.1038/nmicrobiol.2017.64 28452987

[emi470258-bib-0025] Lønborg, C. , K. Davidson , X. A. Álvarez–Salgado , and A. E. J. Miller . 2009. “Bioavailability and Bacterial Degradation Rates of Dissolved Organic Matter in a Temperate Coastal Area During an Annual Cycle.” Marine Chemistry 113: 219–226.

[emi470258-bib-0026] Lønborg, C. , M. Middelboe , and C. P. D. Brussaard . 2013. “Viral Lysis of *Micromonas pusilla* : Impacts on Dissolved Organic Matter Production and Composition.” Biogeochemistry 116: 231–240.

[emi470258-bib-0027] Luef, B. , F. Luef , and P. Peduzzi . 2009. “Online Program ‘Vipcal’ for Calculating Lytic Viral Production and Lysogenic Cells Based on a Viral Reduction Approach.” Environmental Microbiology Reports 1: 78–85. 21151811 10.1111/j.1758-2229.2008.00008.x PMC2999826

[emi470258-bib-0028] Maat, D. S. , M. A. Prins , and C. P. D. Brussaard . 2019. “Sediments From Arctic Tide‐Water Glaciers Remove Coastal Marine Viruses and Delay Host Infection.” Viruses 11: 123. 30704033 10.3390/v11020123 PMC6409924

[emi470258-bib-0029] Maat, D. S. , R. J. W. Visser , and C. P. D. Brussaard . 2019. “Virus Removal by Glacier‐Derived Suspended Fine Sediment in the Arctic.” Journal of Experimental Marine Biology and Ecology 521: 151227.

[emi470258-bib-0030] Marie, D. , F. Partensky , D. Vaulot , and C. Brussaard . 1999. “Enumeration of Phytoplankton, Bacteria, and Viruses in Marine Samples.” Current Protocols in Cytometry 10: 11.11.1–11.11.15. 10.1002/0471142956.cy1111s10 18770685

[emi470258-bib-0031] Marín‐Vindas, C. , M. Sebastián , C. Ruiz‐González , V. Balagué , L. Vega‐Corrales , and J. M. Gasol . 2023. “Shifts in Bacterioplankton Community Structure Between Dry and Wet Seasons in a Tropical Estuary Strongly Affected by Riverine Discharge.” Science of the Total Environment 903: 166104. 37558065 10.1016/j.scitotenv.2023.166104

[emi470258-bib-0032] Meyneng, M. , H. Lemonnier , R. le Gendre , et al. 2024. “Subtropical Coastal Microbiome Variations due to Massive River Runoff After a Cyclonic Event.” Environmental Microbiomes 19: 10. 10.1186/s40793-024-00554-9 PMC10829310 38291506

[emi470258-bib-0033] Middelboe, M. , N. Jorgensen , and N. Kroer . 1996. “Effects of Viruses on Nutrient Turnover and Growth Efficiency of Noninfected Marine Bacterioplankton.” Applied and Environmental Microbiology 62: 1991–1997. 16535334 10.1128/aem.62.6.1991-1997.1996 PMC1388872

[emi470258-bib-0034] Mojica, K. D. A. , and C. P. D. Brussaard . 2020. “Significance of Viral Activity for Regulating Heterotrophic Prokaryote Community Dynamics Along a Meridional Gradient of Stratification in the Northeast Atlantic Ocean.” Viruses 12: 1293. 33198110 10.3390/v12111293 PMC7696675

[emi470258-bib-0035] Mojica, K. , C. Evans , and C. Brussaard . 2014. “Flow Cytometric Enumeration of Marine Viral Populations at Low Abundances.” Aquatic Microbial Ecology 71: 203–209.

[emi470258-bib-0036] Motegi, C. , T. Nagata , T. Miki , M. G. Weinbauer , L. Legendre , and F. Rassoulzadegan . 2013. “Interactive Effects of Viral and Bacterial Production on Marine Bacterial Diversity.” PLoS One 8: e76800. 24244268 10.1371/journal.pone.0076800 PMC3820650

[emi470258-bib-0037] Noble, R. T. , and J. A. Fuhrman . 2000. “Rapid Virus Production and Removal as Measured With Fluorescently Labeled Viruses as Tracers.” Applied and Environmental Microbiology 66: 3790–3797. 10966392 10.1128/aem.66.9.3790-3797.2000 PMC92222

[emi470258-bib-0038] Parada, V. , E. Sintes , H. M. Van Aken , M. G. Weinbauer , and G. J. Herndl . 2007. “Viral Abundance, Decay, and Diversity in the Meso‐ and Bathypelagic Waters of the North Atlantic.” Applied and Environmental Microbiology 73: 4429–4438. 17496133 10.1128/AEM.00029-07 PMC1932831

[emi470258-bib-0039] Parada, V. , G. J. Herndl , and M. G. Weinbauer . 2006. “Viral Burst Size of Heterotrophic Prokaryotes in Aquatic Systems.” Journal of the Marine Biological Association of the United Kingdom 86: 613–621.

[emi470258-bib-0040] Paul, J. H. 2008. “Prophages in Marine Bacteria: Dangerous Molecular Time Bombs or the Key to Survival in the Seas?” ISME Journal 2: 579–589. 18521076 10.1038/ismej.2008.35

[emi470258-bib-0041] Payet, J. P. , and C. A. Suttle . 2013. “To Kill or Not to Kill: The Balance Between Lytic and Lysogenic Viral Infection Is Driven by Trophic Status.” Limnology and Oceanography 58: 465–474.

[emi470258-bib-0042] Racine, J. S. 2012. “Rstudio: A Platform‐Independent Ide for R and Sweave.” Journal of Applied Econometrics 27: 167–172.

[emi470258-bib-0043] Richter, Ł. , K. Księżarczyk , K. Paszkowska , et al. 2021. “Adsorption of Bacteriophages on Polypropylene Labware Affects the Reproducibility of Phage Research.” Scientific Reports 11: 7387. 33795704 10.1038/s41598-021-86571-x PMC8016829

[emi470258-bib-0044] Schwalbach, M. , I. Hewson , and J. Fuhrman . 2004. “Viral Effects on Bacterial Community Composition in Marine Plankton Microcosms.” Aquatic Microbial Ecology 34: 117–127.

[emi470258-bib-0045] Sekiguchi, M. , and Y. Takagi . 1960. “Effect of Mitomycin C on the Synthesis of Bacterial and Viral Deoxyribonucleic Acid.” Biochimica et Biophysica Acta 41: 434–443. 14444659 10.1016/0006-3002(60)90040-8

[emi470258-bib-0046] Shaikh, H. M. , J. den Van Bremt , and S. J. Fernandez . 2023. viralprod R Package: Automating Viral Production Analyses From Viral Production Assays [Version 0.9]. Zenodo. 10.5281/zenodo.14945312.

[emi470258-bib-0047] Shi, W. , and M. Wang . 2010. “Characterization of Global Ocean Turbidity From Moderate Resolution Imaging Spectroradiometer Ocean Color Observations.” Journal of Geophysical Research: Oceans 115: 1–14.

[emi470258-bib-0048] Shruthi, P. , A. Parvathi , A. S. Pradeep Ram , et al. 2022. “Contrasting Impact of Viral Activity on Prokaryotic Populations in the Coastal and Offshore Regions of the Eastern Arabian Sea.” Diversity 14: 230.

[emi470258-bib-0049] Silpe, J. E. , and B. L. Bassler . 2019. “A Host‐Produced Quorum‐Sensing Autoinducer Controls a Phage Lysis‐Lysogeny Decision.” Cell 176: 268–280.e13. 30554875 10.1016/j.cell.2018.10.059 PMC6329655

[emi470258-bib-0050] Strom, S. L. 2008. “Microbial Ecology of Ocean Biogeochemistry: A Community Perspective.” Science 320: 1043–1045. 18497289 10.1126/science.1153527

[emi470258-bib-0051] Suttle, C. A. 2007. “Marine Viruses—Major Players in the Global Ecosystem.” Nature Reviews. Microbiology 5: 801–812. 17853907 10.1038/nrmicro1750

[emi470258-bib-0052] Team, R. C . 2000. R Language Definition. Vol. 3, 116. R Found. Stat. Comput.

[emi470258-bib-0053] Tomasz, M. 1976. “H_2_O_2_ Generation During the Redox Cycle of Mitomycin C and DNA‐Bound Mitomycin C.” Chemico‐Biological Interactions 13: 89–97. 770011 10.1016/0009-2797(76)90016-8

[emi470258-bib-0054] Tomasz, M. 1995. “Mitomycin C: Small, Fast and Deadly (But Very Selective).” Chemistry & Biology 2: 575–579. 9383461 10.1016/1074-5521(95)90120-5

[emi470258-bib-0055] Tuttle, M. J. , and A. Buchan . 2020. “Lysogeny in the Oceans: Lessons From Cultivated Model Systems and a Reanalysis of Its Prevalence.” Environmental Microbiology 22: 4919–4933. 32935433 10.1111/1462-2920.15233

[emi470258-bib-0056] Van Duyl, F. C. , and A. J. Kop . 1988. “Temporal and Lateral Fluctuations in Production and Biomass of Bacterioplankton in the Western Dutch Wadden Sea.” Netherlands Journal of Sea Research 22: 51–68.

[emi470258-bib-0057] Weinbauer, M. , and C. Suttle . 1999. “Lysogeny and Prophage Induction in Coastal and Offshore Bacterial Communities.” Aquatic Microbial Ecology 18: 217–225.

[emi470258-bib-0058] Weinbauer, M. G. , and C. A. Suttle . 1996. “Potential Significance of Lysogeny to Bacteriophage Production and Bacterial Mortality in Coastal Waters of the Gulf of Mexico.” Applied and Environmental Microbiology 62: 4374–4380. 16535459 10.1128/aem.62.12.4374-4380.1996 PMC1388997

[emi470258-bib-0059] Weinbauer, M. , J. Rowe , and S. Wilhelm . 2010. “Determining Rates of Virus Production in Aquatic Systems by the Virus Reduction Approach.” In Manual of Aquatic Viral Ecology, edited by S. Wilhelm , M. Weinbauer , and C. Suttle , 1–8. American Society of Limnology and Oceanography. 10.4319/mave.2010.978-0-9845591-0-7.1.

[emi470258-bib-0060] Weinbauer, M. , C. Winter , and M. Höfle . 2002. “Reconsidering Transmission Electron Microscopy Based Estimates of Viral Infection of Bacterioplankton Using Conversion Factors Derived From Natural Communities.” Aquatic Microbial Ecology 27: 103–110.

[emi470258-bib-0061] Wickham, H. , M. Averick , J. Bryan , et al. 2019. “Welcome to the Tidyverse.” Journal of Open Source Software 4: 1686.

[emi470258-bib-0062] Wilhelm, S. W. , and C. A. Suttle . 1999. “Viruses and Nutrient Cycles in the Sea.” BioScience 49: 781–788.

[emi470258-bib-0063] Wilhelm, S. W. , S. M. Brigden , and C. A. Suttle . 2002. “A Dilution Technique for the Direct Measurement of Viral Production: A Comparison in Stratified and Tidally Mixed Coastal Waters.” Microbial Ecology 43: 168–173. 11984638 10.1007/s00248-001-1021-9

[emi470258-bib-0064] Winget, D. , K. Williamson , R. Helton , and K. Wommack . 2005. “Tangential Flow Diafiltration: An Improved Technique for Estimation of Virioplankton Production.” Aquatic Microbial Ecology 41: 221–232.

[emi470258-bib-0065] Winter, C. , G. Herndl , and M. Weinbauer . 2004. “Diel Cycles in Viral Infection of Bacterioplankton in the North Sea.” Aquatic Microbial Ecology 35: 207–216.

[emi470258-bib-0066] Xie, L. , W. Wei , L. Cai , et al. 2021. “A Global Viral Oceanography Database (gVOD).” Earth System Science Data 13: 1251–1271.

[emi470258-bib-0067] Yi, Y. , S. Liu , Y. Hao , et al. 2023. “A Systematic Analysis of Marine Lysogens and Proviruses.” Nature Communications 14: 6013. 10.1038/s41467-023-41699-4 PMC10533544 37758717

