# Supplementary material for: Refining Viral Production Estimation
Source: Environ Microbiol Rep. 2025 Dec 14;17(6):e70258. doi: 10.1111/1758-2229.70258 (PMC12702599; doi:10.1111/1758-2229.70258)

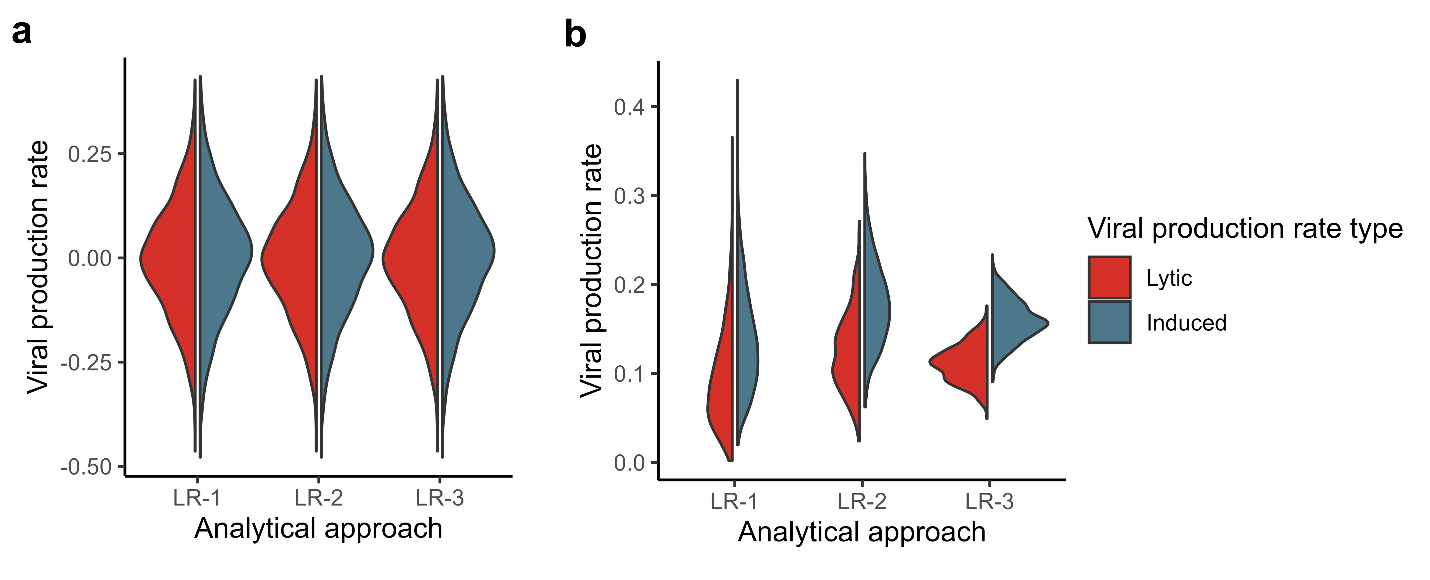


Figure S1. Comparison of 3 variations in the linear regression model (LR) analysis approach in extracting viral production rates. a) No significant differences were observed in lytic and induced viral production estimates between the three variants. b) Significant differences (p < 0.0001) were observed in the standard errors calculated between the three LR variants used to estimate both lytic and induced viral production rates.


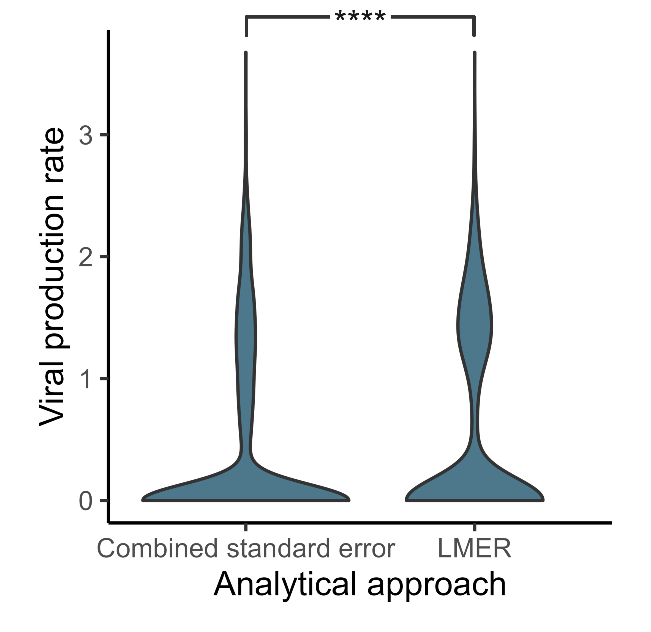


Figure S2. Impact of using combined standard error formula (equation 1) and applying a linear mixed effects model (LMER) to extract standard errors of the difference curve when estimating mitomycin C-induced viral production rates using VIPCAL-SE approach. Significant differences were observed between the two approaches (**** p < 0.0001).


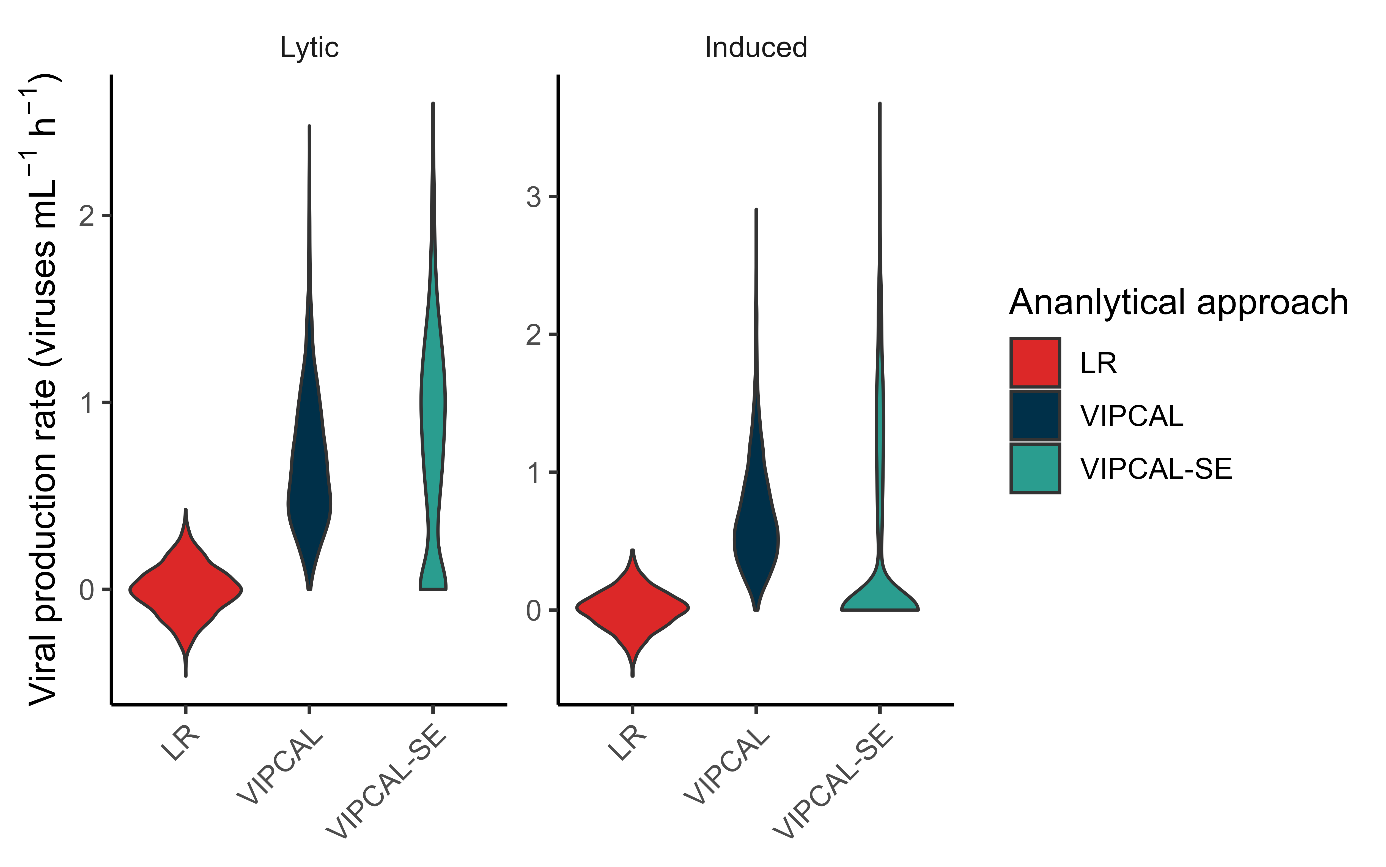


Figure S3. Comparison of lytic and induced viral production rates estimated using LR, VIPCAL, and VIPCAL-SE approaches. All approaches provide significantly different estimates from each other (**** p < 0.0001).

Figure S4. Evolution of mean relative collision rate between bacteria and viruses in VP (no mitomycin C addition) and VPC (mitomycin C) treatments over time in viral production assays performed in coastal North Sea (CNS), open North Sea (ONS), and Curaçao Caribbean Sea (CCS). NGTE is the timepoint at which net bacterial generation time in the VP treatment is less than 24 hours. A divergence in mean relative collision rates were observed between the two treatments after NGTE.


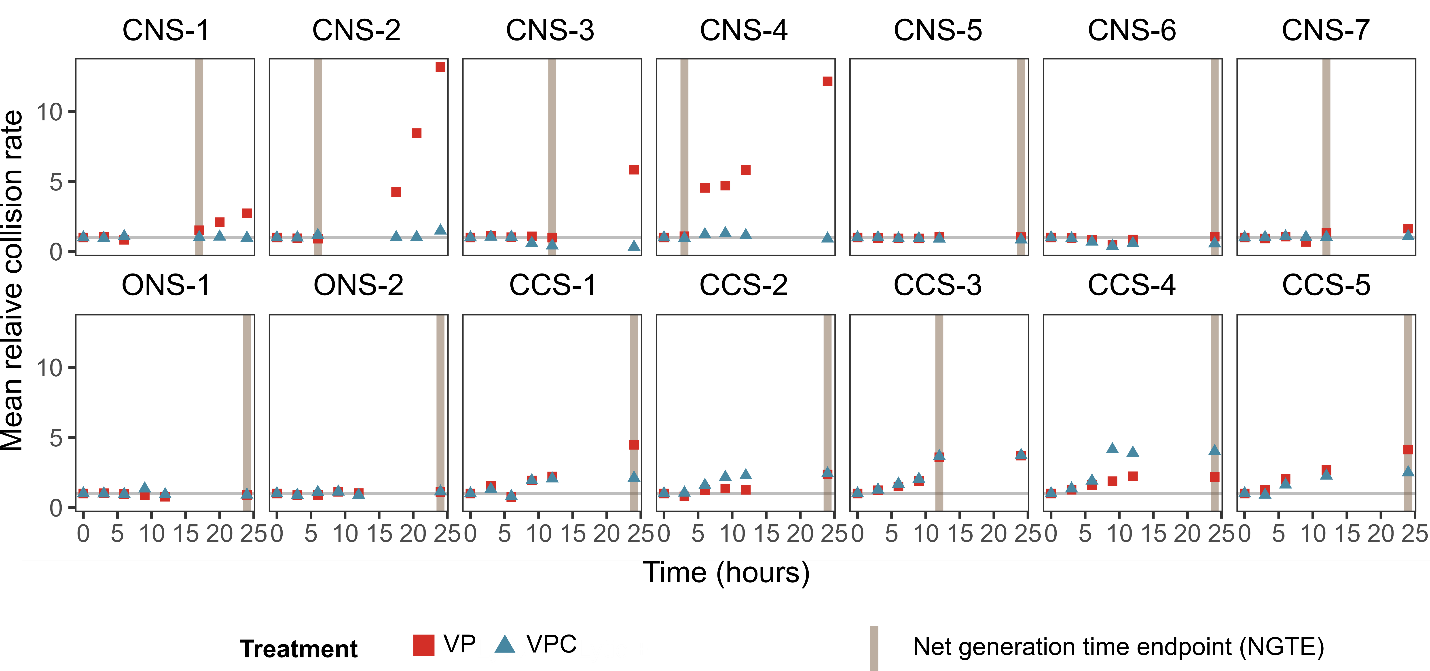

Supplement: Supplementary file 1 — Figure S1: Comparison of three variations in the linear regression model (LR) analysis approach in extracting viral production rates. (a) No significant differences were observed in lytic and induced viral production estimates between the three variants. (b) Significant differences (p < 0.0001) were observed in the standard errors calculated between the three LR variants used to estimate both lytic and induced viral production rates. Figure S2: Impact of using combined standard error formula (Equation 1) and applying a linear mixed effects model (LMER) to extract standard errors of the difference curve when estimating mitomycin C‐induced viral production rates using VIPCAL‐SE approach. Significant differences were observed between the two approaches (****p < 0.0001). Figure S3: Comparison of lytic and induced viral production rates estimated using LR, VIPCAL and VIPCAL‐SE approaches. All approaches provide significantly different estimates from each other (p < 0.0001). Figure S4: Evolution of mean relative collision rate between bacteria and viruses in VP (no mitomycin C addition) and VPC (mitomycin C) treatments over time in viral production assays performed in coastal North Sea (CNS), open North Sea (ONS) and Curaçao Caribbean Sea (CCS). Net generation time endpoint (NGTE) is the timepoint at which net bacterial generation time in the VP treatment is less than 24 h. A divergence in mean relative collision rates were observed between the two treatments after NGTE. [file EMI4-17-e70258-s003.docx]
